# Psychopathology: genetics and the stress-vulnerability hypothesis

**DOI:** 10.1007/s00406-012-0307-x

**Published:** 2012-03-27

**Authors:** Peter Falkai, Hans-Jürgen Möller

**Affiliations:** 1 Department of Psychiatry and Psychotherapy, University of Göttingen, Von-Siebold-Str. 5, 37075 Göttingen, Germany; 2 Department of Psychiatry and Psychotherapy, Ludwigs-Maximililans-University Munich, Nussbaumstr. 7, 80336 Munich, Germany

Since the findings of dopamine blockade by antipsychotics, a dysfunction of the dopamine system has been proposed to underly psychopathological symptoms in schizophrenia. In SPECT and PET imaging studies, radioligand binding to the dopamine receptor or transporter reflects the occupancy by dopamine itself or antipsychotics. Using dual-isotope imaging for the evaluation of pre- and postsynaptic site, Schmitt et al. [1] examined striatal dopamine transporter and dopamine D2 receptor availability in first-episode schizophrenia patients and patients with haloperidol treatment compared to healthy controls. In drug-naïve patients with positive symptoms, dopamine transporter availability correlated negatively with psychopathology as did D2 receptor binding with disorganization and hallucinatory behavior, thus strengthening the dopamine hypothesis for positive symptoms. D2 receptor and dopamine transporter availability was lower in the haloperidol group which confirms previous dopamine blockade studies. However, response to antipsychotics is individual, and genetic factors may contribute to a different outcome. Mössner et al. [2] reported that patients homozygous for the risk allele ZNF804A show poorer improvement of positive symptoms compared to patients with a protective allele. Thus, identification of risk genes may help identifying patients with a different course of the disorder. A pharmacogenetic study of Crisafulli et al. [3] investigated 10 SNPs of genes related to pathophysiological pathways in Korean patients. They found a SNP within 5HTR1A associated with clinical improvement in both positive and negative symptoms.

In schizophrenia patients, improvement of quality of life as treatment outcome among others is dependent on support in case of problems and assistance in coping with daily life demands. Landolt et al. [4] revealed that unmet needs changing to fulfilled ones enhanced quality of life. In this context, psychosocial functioning and psychotic symptoms may be influenced by alterations of theory of mind and empathy as underlying social-cognitive deficits and possibly are heritable. In first-degree relatives of schizophrenia patients, Montag et al. [5] showed impairment in cognitive skills, suggesting that social-cognitive abilities may serve as an intermediate phenotype for schizophrenia. In a re-analysis of AMDP data by nonmetric multidimensional scaling, Läge et al. [6] replicated clusters of psychopathological symptoms in two independent samples dating from 1980 and 2003. In individual’s health, the psychological well-being dimension and depressive symptoms are important variables and Morelatto de Souza and Paz Loayza Hidalgo [7] validated the WHO well-being index in a Brazilian population and showed this scale to be a useful tool for screening of depressive symptoms. Hagihara and Abe [8] highlight the influence of media reports on suicide and counteracting effects of stopping the sale of suicide-related products in Japan.

Beside genetics, stressful experience and trauma are risk factors for the development of psychiatric diseases. Linden et al. [9] analyzed 100 case files of German servicemen during World War I and classified them in agreement with modern diagnoses. Most ancient diagnoses were “psychopathic constitution” or “hysteria.” Using contemporary diagnostics, some of these soldiers had symptoms of post-traumatic stress disorder. There was a high incidence of pseudoseizures that highlights different human responses to traumatic experience and stress in a historical context. Stressful experiences are known to increase vulnerability to schizophrenia. Rossi et al. [10] evaluated the Community Assessment of Psychic Experiences (CAPE) in students who survived an earthquake 2009 in Italy. Unexpectedly, earth quake survivors showed lower CAPE scores than nonexposed subjects. This is in line with the hypothesis that stressful events preceding psychosis are not derived from external traumatic experiences. In a previous study, Puustinen et al. [11] reported C-reactive protein, marker of cardiovascular disease, to be associated with psychological distress. However, in a cross-sectional and 3-year follow-up study in Japanese male workers, Kawada [12] failed to show this relationship, which is probably due to ethnicity. Life experience, stressful events and other environmental factors are known to influence the risk of developing mental diseases. The missing link between environment and neurobiology may be explained by epigenetic mechanisms, such as DNA methylation and acetylation in disease-related brain regions. Wiers [13] refers to an fMRI study of the COMT gene with the Val allele being associated with DNA methylation [14]. She proposes the use of genetic imaging in combination with investigations of epigenetic markers in blood cells to unravel gene-environmental interactions in severe psychiatric disorders.
